# Supplementary material for: From alternative conceptions of honesty to alternative facts in communications by US politicians
Source: Nat Hum Behav. Author manuscript; Available in PMC 2023 Dec 22. (PMC10730411; doi:10.1038/s41562-023-01691-w)
Supplement: Supplementary Information [file EMS191278-supplement-Supplementary_Information.pdf]

nature human behaviour

Article https://doi.org/10.1038/s41562-023-01691-w

From alternative conceptions of honesty to alternative facts in communications by US politicians

In the format provided by the authors and unedited

S1 Instructions to participants during keyword validation

What follows is a verbatim copy of the instructions provided to the participants who rated the keywords. Note that in the original version of the instructions, "fact-speaking" was called "truth-seeking". We have revised the nomenclature throughout this work to clarify the concept.

People can have different ideas about what it means to be "honest".

We are focusing on two ideas of honesty.

One is based on intuition, "gut feeling" and authenticity. According to this idea, people speak the truth and are honest when they "say what they felt to be true in the moment". Whether or not claims are correct reflections of reality is not as important. We call this idea of honesty and truth "belief speaking".

The other idea is based on evidence, analysis, and veracity. According to this idea, people speak the truth and are honest when their claims align with the evidence. Whether or not claims are authentic reflections of a person's feelings is not as important. We call this idea of honesty and truth "fact-speaking".

Your task is to judge, for each of the words below, which idea of honesty it is most closely related to. If someone uses that word, does it likely reflect belief speaking? Or does the word likely reflect fact-speaking?

Please indicate which idea of honesty each word is closest to by selecting, for each column, a value from 1 to 5, where 1 means that the word is the least representative of that category, and 5 means that the word is highly representative of that category. There are no right or wrong answers, we are interested in your analysis of the meaning of those words.

S2 Dictionary keyword validation results

To validate the keywords contained in the belief-speaking and fact-speaking dictionaries we asked raters on the survey platform Prolific[1] to score each term on two scales reflecting their representativeness for belief-speaking and fact-speaking, respectively. The collected data contains responses from 50 participants and ratings from 1 to 5 for each keyword. Data were acquired September 20, 2022, the instructions provided to participants are reported in section "Prolific Questionnaire Instructions". The distributions of ratings collected for each keyword are shown in Figures Supplementary Figure 1 and Supplementary Figure 2.

To determine the validity of each keyword, we conducted t-tests between the distribution of representativeness ratings for belief-speaking and the distribution of representativeness ratings for fact-speaking for every keyword. If the difference between the distributions was significant ($\alpha = 0.05$), the keyword was included in the belief-speaking dictionary if the t-value was positive, and in the fact-speaking dictionary if the t-value was negative. Results of the t-tests for each keyword are reported in Table Supplementary Table 1.

Supplementary Table 1: Results of the two-sided paired t-tests of the keyword ratings performed by 50 raters. "component" indicates the honesty component a given keyword was initially assigned to. The column "valid" is a binary variable indicating whether our initial component assignment for the keyword was confirmed by the raters, based on the t-value direction (positive for belief-speaking, negative for fact-speaking) and a significance level of $\alpha = 0.05$. The column "opposite" indicates whether a keyword was shifted to the opposite honesty component dictionary. This happened when the t-value was significant ($\alpha = 0.05$) but in the opposite direction than initially assumed. Rating distributions are shown in Figure Supplementary Figure 1 for the keywords that were initially categorised as "belief-speaking" and in Figure Supplementary Figure 2 for the keywords that were initially categorised as "fact-speaking".

keyword	t value	p value	component	valid	opposite
actually	-3.7939	0.0004	fact	yes	no
admittedly	1.2102	0.2318	belief	no	no
analyze	-11.8607	0.0000	fact	yes	no
assert	1.6488	0.1053	fact	no	no
assertion	1.9003	0.0630	fact	no	no
assess	-6.6167	0.0000	fact	yes	no
basically	5.6661	0.0000	belief	yes	no
believe	12.9276	0.0000	belief	yes	no
certainly	-1.7321	0.0893	belief	no	no
claim	3.7398	0.0005	fact	no	yes
clearly	0.2989	0.7663	belief	no	no
confide	5.5550	0.0000	belief	yes	no
consider	2.6606	0.0104	belief	yes	no
contemplate	3.3981	0.0013	fact	no	yes
contention	2.0449	0.0460	fact	no	yes
correct	-4.5756	0.0000	fact	yes	no
correction	-4.7842	0.0000	fact	yes	no
definitely	-1.8134	0.0757	belief	no	no
determine	-5.4070	0.0000	fact	yes	no
doubtless	-0.5534	0.5824	belief	no	no
envisage	5.5751	0.0000	belief	yes	no
estimate	1.6797	0.0991	fact	no	no
evaluate	-9.2428	0.0000	fact	yes	no
evidence	-13.5218	0.0000	fact	yes	no
examine	-7.2276	0.0000	fact	yes	no
exploration	-3.7341	0.0005	fact	yes	no
explore	-1.4402	0.1559	fact	no	no
fact	-14.9015	0.0000	fact	yes	no
feel	13.3212	0.0000	belief	yes	no
find	-1.9767	0.0535	fact	no	no
frankly	5.3732	0.0000	belief	yes	no
genuinely	2.1898	0.0331	fact	no	yes
guess	11.8937	0.0000	belief	yes	no
hint	3.9430	0.0002	fact	no	yes
honestly	1.0163	0.3143	belief	no	no
improvement	-1.0674	0.2908	fact	no	no
indeed	-0.5380	0.5929	belief	no	no
information	-7.8184	0.0000	fact	yes	no
inspect	-8.3901	0.0000	fact	yes	no
investigate	-10.0865	0.0000	fact	yes	no
judge	4.4555	0.0000	fact	no	yes
look	2.1598	0.0355	fact	no	yes
no doubt	0.8743	0.3860	belief	no	no
observe	-4.3294	0.0001	belief	no	yes
obvious	2.7386	0.0085	belief	yes	no
obviously	4.6009	0.0000	belief	yes	no
of course	3.9459	0.0002	belief	yes	no
opinion	15.1750	0.0000	belief	yes	no
overhaul	1.1481	0.2563	fact	no	no
plainly	2.5317	0.0145	belief	yes	no
ponder	4.9805	0.0000	fact	no	yes
position	2.0462	0.0459	belief	yes	no
presume	8.7004	0.0000	belief	yes	no

probably	5.7093	0.0000	belief	yes	no
proof	-12.3100	0.0000	fact	yes	no
prove	-8.3425	0.0000	fact	yes	no
question	-3.2428	0.0021	fact	yes	no
quiz	-4.4351	0.0000	fact	yes	no
rate	-1.1864	0.2409	fact	no	no
real	-4.3970	0.0001	fact	yes	no
reality	-5.6908	0.0000	fact	yes	no
really	1.0758	0.2871	belief	no	no
rectify	-2.5995	0.0122	fact	yes	no
reflect	1.9660	0.0548	fact	no	no
research	-10.5963	0.0000	fact	yes	no
revise	-4.6863	0.0000	fact	yes	no
sample	-7.0234	0.0000	fact	yes	no
science	-12.6170	0.0000	fact	yes	no
scrutinize	-2.1898	0.0331	fact	yes	no
search	-3.7338	0.0005	fact	yes	no
seem	8.6065	0.0000	belief	yes	no
sensation	11.1959	0.0000	belief	yes	no
sentiment	11.0784	0.0000	belief	yes	no
signal	2.5428	0.0141	fact	no	yes
specify	-5.4430	0.0000	fact	yes	no
suggest	5.1004	0.0000	fact	no	yes
suggestion	6.6150	0.0000	belief	yes	no
supervise	-2.5412	0.0141	fact	yes	no
suppose	8.6284	0.0000	belief	yes	no
sure	0.3841	0.7025	belief	no	no
surely	3.0461	0.0037	belief	yes	no
tentative	1.6450	0.1061	fact	no	no
test	-9.2804	0.0000	fact	yes	no
testimony	0.1301	0.8970	fact	no	no
think	4.3846	0.0001	belief	yes	no
trace	-2.7584	0.0080	fact	yes	no
track	-8.4954	0.0000	fact	yes	no
trial	-6.3374	0.0000	fact	yes	no
truly	1.1579	0.2523	belief	no	no
trust	2.4280	0.0187	belief	yes	no
truth	-4.9316	0.0000	fact	yes	no
try	3.0329	0.0038	fact	no	yes
undoubtedly	-0.3104	0.7575	belief	no	no
validate	-8.0957	0.0000	fact	yes	no
verify	-15.4471	0.0000	fact	yes	no
view	5.0381	0.0000	belief	yes	no
virtually	2.6190	0.0116	fact	no	yes
witness	-0.8494	0.3996	fact	no	no

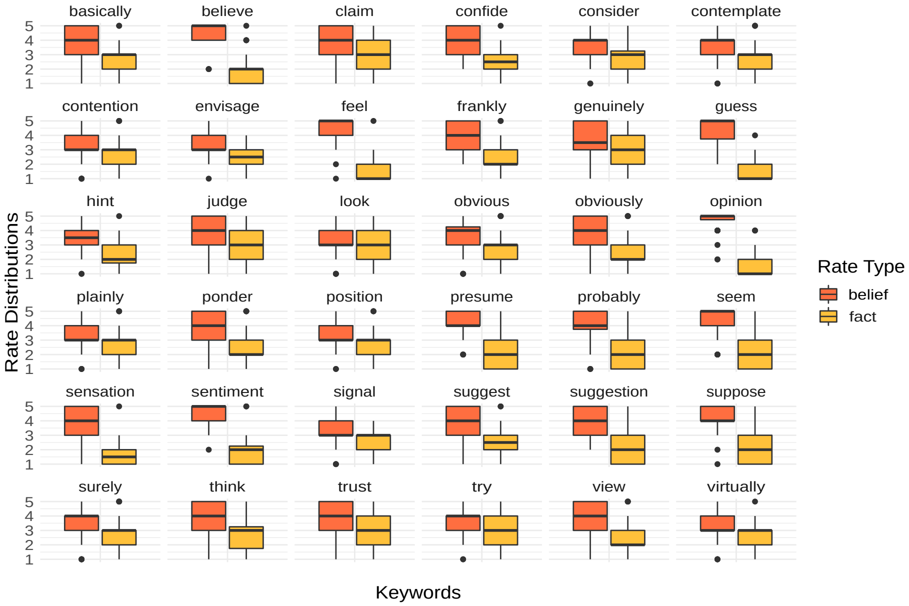

Supplementary Figure 1 Boxplots of rating distributions for keywords we originally categorized as belief-speaking. Clear cases where our categorizations were confirmed are, for example, 'opinion', 'feel', 'believe'. Examples of discarded keywords are 'clearly', 'undoubtedly', 'sure'. The only reversed case is 'observe', categorized as 'fact-speaking' by the raters. The horizontal lines indicate the mean over N=50 observations, boxes extend from the first to third quartile and whiskers indicate 1.5 IQR.

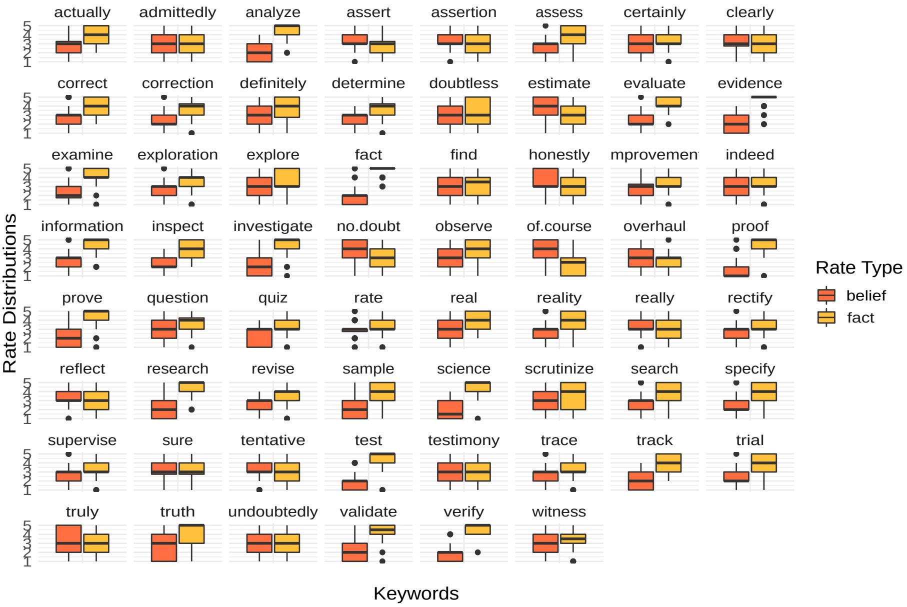

Supplementary Figure 2 Boxplots of rating distributions for keywords we originally categorized as fact-speaking. Clear cases where our categorizations were confirmed are, for example, 'verify', 'fact', 'evidence'. Examples of discarded keywords are 'witness', 'testimony', 'overhaul'. Instances of reversed keywords are 'suggest', 'ponder', 'judge', categorized as 'belief-speaking' by the raters. The horizontal lines indicate the mean over N=50 observations, boxes extend from the first to third quartile and whiskers indicate 1.5 IQR.

S3 Document-level validation results

To validate the belief-speaking and fact-speaking measures, we asked raters on the survey platform Prolific[1] to score tweets on two scales reflecting their representativeness for belief-speaking and fact-speaking, respectively.

Tweets shown to the participants were sampled from the full corpus of tweets with the aim of sampling tweets with high and low honesty component similarity D'_{b} and D'_{f}. We thus sampled 20 tweets from the top belief-speaking and bottom fact-speaking quartile, as well as 20 tweets from the top fact-speaking and bottom belief-speaking quartile. In addition, we sampled 20 tweets that simultaneously belonged to the bottom belief-speaking and fact-speaking quartiles. Each sample of 20 tweets included 10 tweets from Democrats and 10 from Republicans.

The collected data contains responses from 50 participants (one participant from the initial 51 participants was excluded due to failing the attention check) and ratings from 1 to 5 for each tweet for belief-speaking and fact-speaking, respectively. Data were acquired February 10, 2023. The instructions provided to participants are the same as those reported in Section S1 with the only adaptation that the term "word" was replaced with the term "tweet".

We then classified every tweet for which a majority of human raters selected either a "4" or a "5" for how characteristic a tweet was for "belief-speaking" ["fact-speaking"] as "belief-speaking" ["fact-speaking"] to create a ground-truth dataset to compare our classifier against. This resulted in 27 tweets that were classified as "belief-speaking", 21 tweets that were classified as "fact-speaking" and 12 tweets that were classified as neither by human raters.

To assess the performance of our similarity-based classifier, we calculate the ROC curves for belief-speaking as the threshold for the belief-speaking similarity D'_{b} to classify a tweet as "belief-speaking" is varied (see Figure Supplementary Figure 3, left panel). The ROC curve for the fact-speaking similarity D'_{f} is shown in the right panel of Figure Supplementary Figure 3. The area under the curve is high in both cases, with AUC = 0.824 for belief-speaking and AUC = 0.772 for fact-speaking. The distributions of ratings collected for each keyword are shown in Figures Supplementary Figure 4, Supplementary Figure 5 and Supplementary Figure 6.

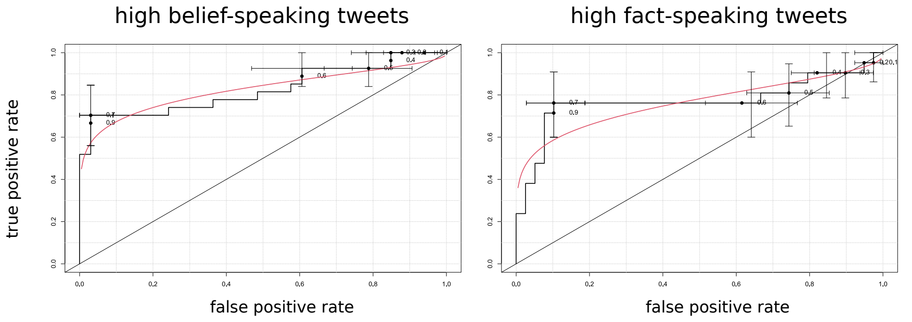

Supplementary Figure 3 ROC curves for the classification of individual tweets into belief-speaking (left) and fact-speaking (right) using ratings from N=50 respondents. Error bars indicate 95% confidence intervals calculated by bootstrapping the observations and predictions.

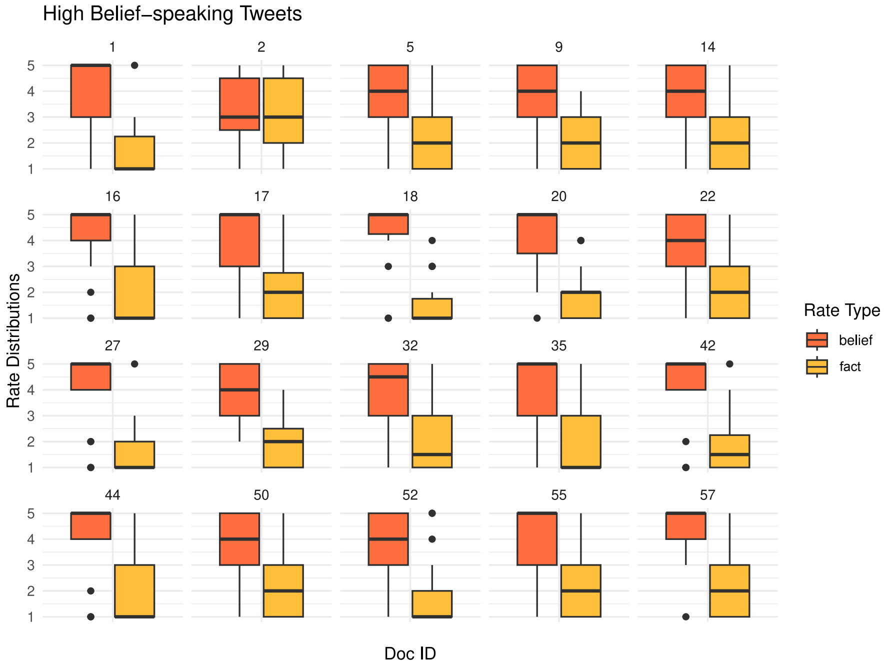

Supplementary Figure 4 Boxplots of rating distributions for tweets sampled from the top belief-speaking and bottom fact-speaking quantiles. The horizontal lines indicate the mean over N=50 observations, boxes extend from the first to third quartile and whiskers indicate 1.5 IQR.

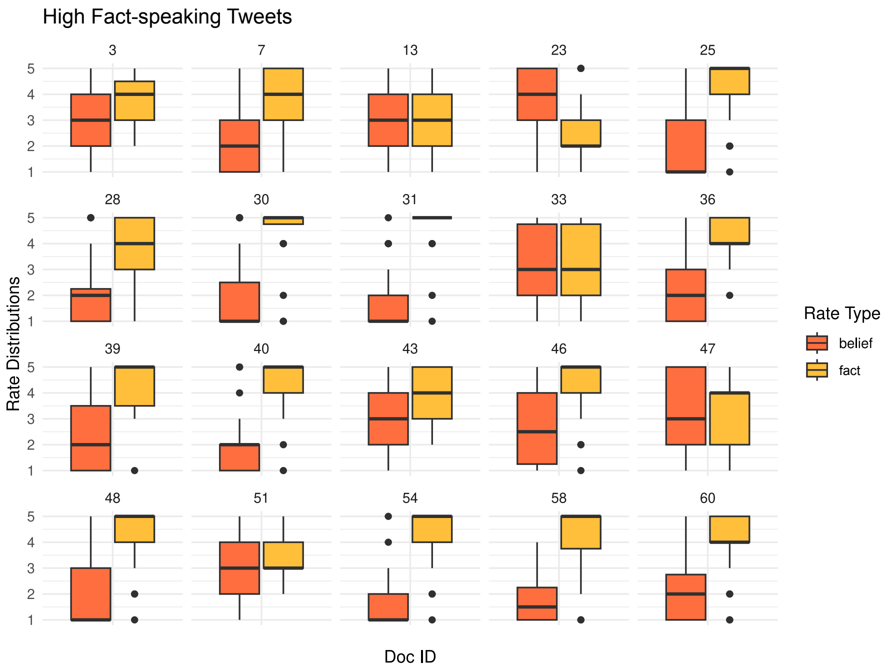

Supplementary Figure 5 Boxplots of rating distributions for tweets sampled from the top fact-speaking and bottom belief-speaking quantiles. The horizontal lines indicate the mean over N=50 observations, boxes extend from the first to third quartile and whiskers indicate 1.5 IQR.

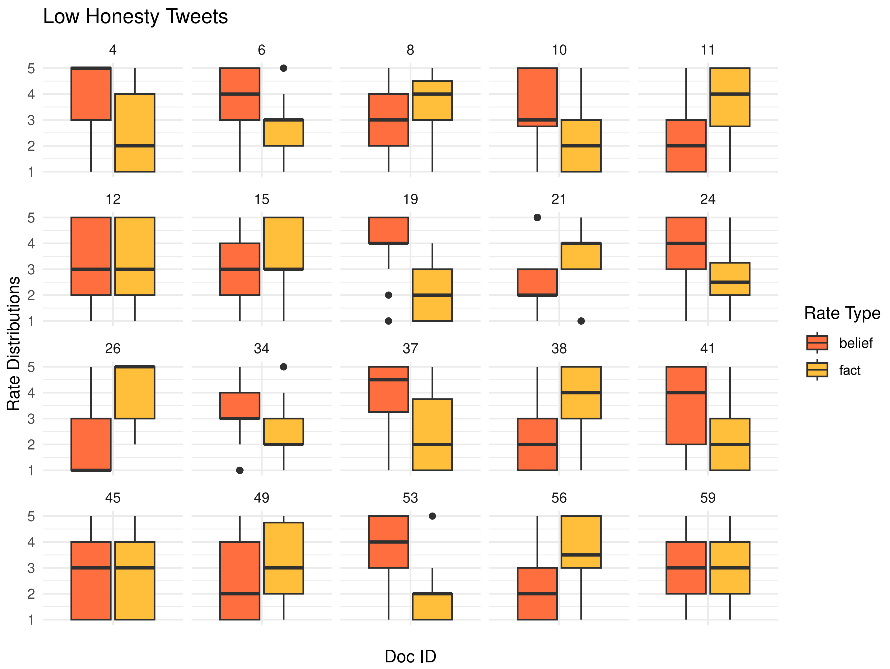

Supplementary Figure 6 Boxplots of rating distributions for tweets sampled from the bottom belief-speaking and fact-speaking quantiles. The horizontal lines indicate the mean over N=50 observations, boxes extend from the first to third quartile and whiskers indicate 1.5 IQR.

S4 VADER text analysis

We explored the content of the tweet texts within the two honesty components using Valence Aware Dictionary for sEntiment Reasoning (VADER) [2]. VADER is a lexicon and rule-based sentiment analysis tool that is specifically attuned to sentiments expressed in social media. VADER computes sentiment polarity of a text and provides a "positive" and "negative" sentiment score, as well as a "neutral" and "compound" score.

Correlations between VADER scores and belief-speaking and fact-speaking similarity are given in Table Supplementary Table 2. In addition, we show the time-development of the positive and negative scores broken down for the top and bottom quantiles of belief-speaking and fact-speaking similarity in Figure Supplementary Figure 7.

Supplementary Table 2 Pearson correlation between belief-speaking and fact-speaking similarity and LIWC scores measuring the prevalence of "analytic", "authentic" and "moral" language, as well as positive and negative sentiment measured with VADER.

Honesty component	Analytic	Authentic	Moral	Pos. sentiment	Neg. sentiment
Belief-speaking	-0.27	0.10	0.07	0.06	0.19
Fact-speaking	-0.16	0.06	0.02	-0.01	0.15

S5 LIWC text analysis

We also explored the content of the tweet texts within the two honesty components using the Linguistic Inquiry and Word Count (LIWC) program [3]. LIWC is a text processing software that has been continuously developed for more than two decades and computes several indicator variables from text based on word lists generated by psychologists and validated in various experiments — similar to our approach in generating the word lists for the belief-speaking and fact-speaking word lists.

With the Beta version of LIWC-2022 software (https://www.liwc.app/), we computed the scores for each tweet text for the following LIWC categories: authenticity, analytic, and moral. Authenticity indicates to what extent the language used is perceived as honest and genuine [4]. Analytic is linked to logical and formal thinking [5]. Finally, moral reflects the judgmental language expressed by positive or negative evaluation of someone's behavior or character [6]. The scores provide an efficient summary of those attributes in each text.

Correlations between LIWC scores and belief-speaking and fact-speaking similarity are given in Table Supplementary Table 2. In addition, we show the time-development of the scores broken down for the top and bottom quantiles of belief-speaking and fact-speaking similarity for the "analytic", "authentic" and "moral" components in Figure Supplementary Figure 8.

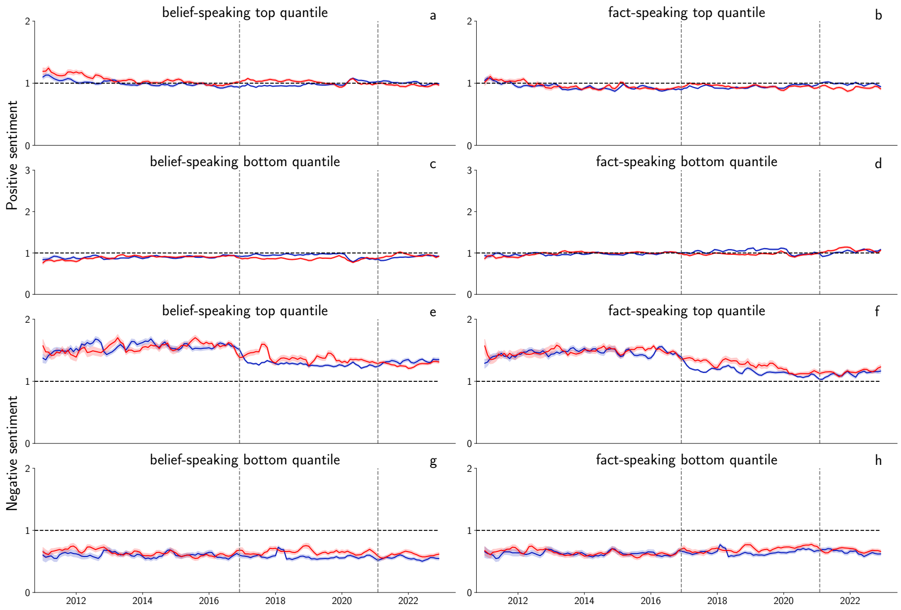

Supplementary Figure 7 Time-development of VADER scores of positive and negative sentiment in tweets of members of the U.S. Congress. Panels a and b show the score for positive sentiment for tweets that belong to the top belief-speaking and fact-speaking similarity quantile, while panels c and d show the positive sentiment score for the bottom similarity quantiles. Timelines are normalized by the overall sentiment score (baseline) for positive sentiment measured in the full corpus. The dashed horizontal line at 1.0 corresponds to prevalence equal to baseline. Red and blue lines correspond to tweets by Republicans and Democrats, respectively. Panels e, f, g and h show the same information as panels a, b, c and d, but for the negative sentiment score instead of the positive sentiment score. The 95% confidence intervals (indicated by shading) were computed with bootstrap sampling over 1,000 iterations. Dashed vertical lines indicate dates of presidential elections in 2016 and 2020. Timelines are smoothed, using a rolling average over three months.

Figure Supplementary Figure 7 shows the timelines of LIWC scores for positive and negative emotions for the top and bottom quantile for belief-speaking and fact-speaking similarity. We performed the same analysis for “authentic”, “analytic” and “moral” language, using LIWC dictionaries as described in the Methods Section “LIWC text analysis”. The time development of “analytic” language broken down by honesty component is shown in Figure Supplementary Figure 8, panels **A** to **D**, the time development of “authentic” language is shown in Figure Supplementary Figure 8 panels **E** to **H** and the time development of “moral” language is shown in Figure Supplementary Figure 8 panels **I** to **L**.

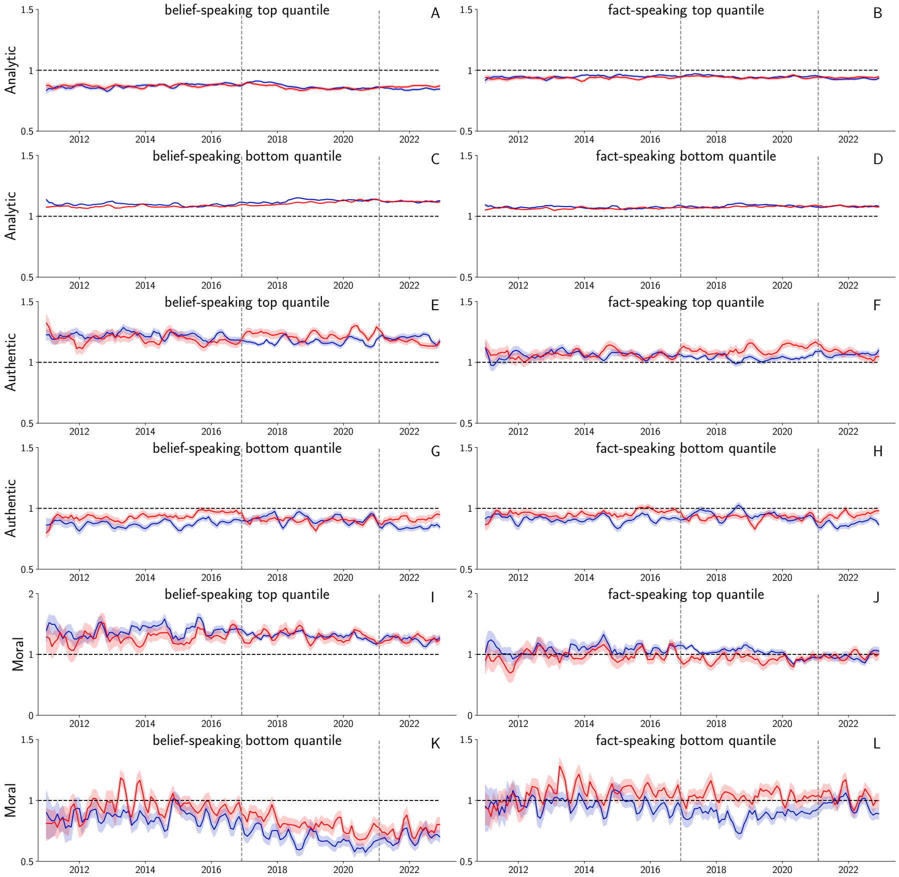

Supplementary Figure 8 Time-development of LIWC scores of “analytic”, “authentic” and “moral” language in tweets of members of the U.S. Congress. Panels a and b show the “analytic” score for tweets that belong to the top belief-speaking and fact-speaking similarity quantile, while panels c and d show the “analytic” score for the bottom similarity quantiles. Timelines are normalized by the overall “analytic” score (baseline) measured in the full corpus. Red and blue lines correspond to tweets by Republicans and Democrats, respectively. Panels e to h show the same information as panels a to d, but for “authentic” language, while panels i to l show the same information for “moral” language. The 95% confidence intervals (indicated by shading) were computed with bootstrap sampling over 1,000 iterations. Dashed vertical lines indicate dates of presidential elections in 2016 and 2020. Timelines are smoothed, using a rolling average over three months.

S6 Topic analysis

To investigate the prevalence of belief-speaking and fact-speaking, we performed topic modelling using the Python package BERTopic[7]. Following a three-step approach, the package uses the Sentence-BERT (SBERT) framework to create the embeddings for each document, then uses the Uniform Manifold Approximation and Projection (UMAP) technique[8] to decrease the dimensionality of embeddings and identify clusters through HDBSCAN[9]. Finally, it creates topic representations using class-based term-frequency inverse-document-frequency (TF-IDF). We opted for BERTopic rather than other techniques such as Latent Dirichlet Allocation (LDA) because the former performs better when modelling short and unstructured texts as in the case of Twitter data when compared to the latter[10;11]. Since BERTopic relies on an embedding approach, data was only minimally preprocessed to keep the original sentence structure. This means we lemmatized the entire dataset to produce cleaner topic representations, and only removed URLs from the texts.

Since the number of documents was too large to fit a topic model of all documents, we restricted the corpus to the last 3200 tweets from each account. We also applied thresholds to the topic modelling: The document minimum frequency was set to 200 in order to reduce the number of small topics. The number of neighboring sample points used when making the manifold approximation was set to 100 to produce a more global view of the embedding structure. Finally, the minimum document frequency for the c-TF-IDF was set to 50 to reduce the topic-term matrix size and decrease memory-related issues during the computation. With these settings, the model was able to identify 363 topics.

To check whether this was an optimal number of topics, we used ldatuning[12], an R package that trains multiple models and calculates validation metrics. Despite the fact that *ldatuning* does not employ embeddings but Latent Dirichlet allocation and that the data it modelled was preprocessed by removing stopwords and irrelevant text (numbers, unknown characters, URLs, Twitter handles), it indicated 300 as an optimal number of topics for the dataset, thus converging towards the BERTopic results.

Building on the topic modelling, we investigated the difference between belief-speaking and fact-speaking in communication about controversial topics in U.S. politics, such as foreign policy, climate change, or the death penalty, and how this differs by party. The selection of controversial topics presented here is inspired by other research in the same area, e.g.[13] and current research topics of non-partisan think-tanks, e.g.[14]. By default, BERTopic assigns each document to a single topic. Therefore, we used this information to calculate how particular controversial topics were distributed across parties and components, as shown in Figure Supplementary Figure 9. To do this, we grouped the tweets by the topic they were assigned to as well as by the party the

politician that created them was affiliated with. We then averaged their belief-speaking and fact-speaking similarity scores to calculate $\langle D'_{\mathrm{b}} \rangle_{\text{topic, party}}$ and $\langle D'_{\mathrm{f}} \rangle_{\text{topic, party}}$, respectively. We repeated this procedure for all 20 topics of interest. We also calculated the average belief-speaking similarity score $\langle D'_{\mathrm{b}} \rangle$ and fact-speaking similarity score $\langle D'_{\mathrm{f}} \rangle$ for all 363 topics found by BERTopic. Finally, we subtracted the specific component averages of a topic t from the full corpus component averages to highlight how parties differ in honesty-speech when talking about controversial matters.

In Figure Supplementary Figure 9 **A** and **B** we show the average belief-speaking and fact-speaking similarity within a given topic $\langle D'_{\mathrm{b}} \rangle_{\text{topic, party}}$ and $\langle D'_{\mathrm{f}} \rangle_{\text{topic, party}}$, minus the average belief-speaking and fact-speaking similarity calculated over the full corpus $\langle D'_{\mathrm{b}} \rangle$ and $\langle D'_{\mathrm{f}} \rangle$ for members of the Democratic and Republican parties, respectively. Each horizontal bar in the figure thus represents the deviation from the average score across the entire corpus. A value greater than zero implies that a topic involved more belief-speaking or fact-speaking than expected on average, and a value less than zero implies below-average invocation of belief-speaking or fact-speaking. It is immediately apparent that most of these controversial topics invoked more belief-speaking or fact-speaking than the average tweet, with only a few exceptions. For example, vaccine related discourse involved far less belief-speaking than any other topic for both parties.

There is, however, also considerable heterogeneity in the amount of belief-speaking and fact-speaking used between the topics: Topics such as impeachment, religious freedom and Putin / Ukraine show a large amount of belief-speaking in both parties, whereas topics such as vaccines show little. Similarly, for fact-speaking the topics climate change, impeachment and religious freedom show a large share of this honesty component for both parties whereas the LGBTQ topic shows little.

There are also marked differences in the balance of belief-speaking and fact-speaking within a topic and between the parties. The topics of climate change, gun violence, COVID-19 and the gender pay gap have the largest difference in belief-speaking, with tweets by Democrats containing more belief-speaking than those by Republicans. The topics of climate change, police, Afghanistan and abortion have the largest difference in fact-speaking with tweets by Democrats containing more fact-speaking while for the topic of animal cruelty, tweets by Republicans contain more fact-speaking.

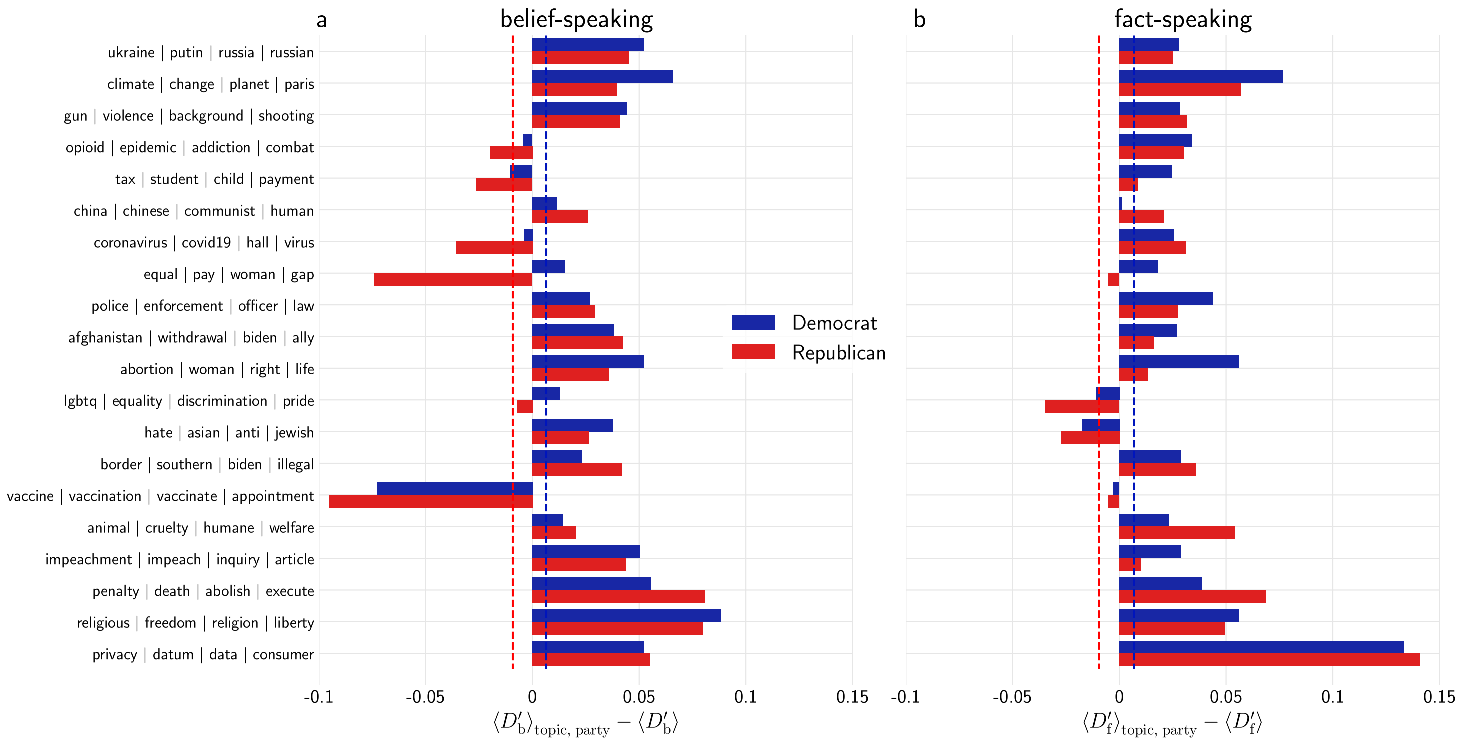

Supplementary Figure 9 Panels a and b show the difference between within-topic within-party belief-speaking and fact-speaking similarity $\langle D'_{\mathrm{b}}\rangle_{\text{topic, party}}$ and $\langle D'_{\mathrm{f}}\rangle_{\text{topic, party}}$ and mean corpus belief-speaking and fact-speaking similarity $\langle D'_{\mathrm{b}}\rangle$ and $\langle D'_{\mathrm{f}}\rangle$, respectively for a range of hand-picked controversial topics. Values for Democrats and Republicans correspond to blue and red bars, respectively. Dashed lines indicate the mean belief-speaking and fact-speaking similarity for each party in the full corpus $\langle D'_{\mathrm{b}}\rangle_{\text{party}} - \langle D'_{\mathrm{b}}\rangle$ and $\langle D'_{\mathrm{f}}\rangle_{\text{party}} - \langle D'_{\mathrm{f}}\rangle$, respectively.

S7 Validation using an independently compiled list of unreliable news sources

To exclude a dependence of the main results reported in Section "Relation of honesty components to information trustworthiness" on use of the News-Guard data base, we validated this analysis with an independently collected list of news outlet reliability from academic and fact-checking sources. Details on how this list was compiled are reported in Section "Independent list of untrustworthy sources" below. Using this list, we can assign an accuracy score S_{a} ranging from 1 to 5 as well as a transparency score S_{t}, ranging from 1 to 3 to each domain. In addition, a domain with an accuracy score of ≤ 2 and/or a transparency score of 1 will be labelled as "unreliable". Similar to the analysis above, we analyse the dependency of the accuracy score S'_{a} rescaled to [0; 1] and the transparency score S'_{t} rescaled to [0; 1] on the centered and length-corrected belief-speaking and fact-speaking similarity measured in tweet texts D'_{b} and D'_{f}, respectively. We fit a linear mixed effects model with party as fixed variable and random slopes and intercepts for every Congress Member for each of the two scores:

$$S'_{\mathrm{a}} \sim 1 + D'_{\mathrm{b}} \times D'_{\mathrm{f}} + D'_{\mathrm{b}} \times D'_{\mathrm{f}} \times P + (1 + D'_{\mathrm{b}} \times D'_{\mathrm{f}} \mid \mathrm{userID}) \quad (1)$$

$$S'_{\mathrm{t}} \sim 1 + D'_{\mathrm{b}} \times D'_{\mathrm{f}} + D'_{\mathrm{b}} \times D'_{\mathrm{f}} \times P + (1 + D'_{\mathrm{b}} \times D'_{\mathrm{f}} \mid \mathrm{userID}) \quad (2)$$

Again, we found a significant positive fixed effect of D'_{f} ($t(442, 500) = 10.7$, $p < 0.001$, coefficient 0.097, 95% CI [0.079; 0.115]) and accuracy S'_{a} as well as for party P = Republican ($t(442, 500) = -18.8$, $p < 0.001$, coefficient -0.071 [-0.079; -0.064]). We reproduce the negative effect of the interaction term between D'_{b} and Republican ($t(442, 500) = -4.4$, $p < 0.001$, coefficient -0.059 [-0.085; -0.033]), the interaction term between D'_{f} and Republican ($t(442, 500) = 2.9$, $p < 0.001$, coefficient 0.040 [0.013; 0.067]), and the three-way interaction between D'_{b}, D'_{f} and Republican ($t(442, 500) = -7.3$, $p < 0.001$, coefficient -0.196 [-0.249; -0.144]).

Different from the main analysis, we also find a significant negative effect for D'_{b} ($t = -13(442, 500).6$, $p < 0.001$, coefficient -0.120 [-0.137; -0.103]).

We see the same pattern for the transparency score S'_{t}, where we see a significant negative relation with D'_{b} and a significant positive relation with D'_{f} for both parties, as well as a significant effect of party, the interaction terms party $\times D'_{\mathrm{b}}$ and party $\times D'_{\mathrm{f}}$, and three-way interaction $D'_{\mathrm{b}} \times D'_{\mathrm{f}} \times$ party.

Full regression statistics are reported in Tables Supplementary Table 3 and Supplementary Table 4. We note that there is extensive agreement between the trustworthiness labels in the NewsGuard data base and the alternative data base: An account that is labelled "untrustworthy" in the NewsGuard data base has a high chance of being labelled "unreliable" in the alternative

Supplementary Table 3 Results of a linear mixed effects model for the dependence of the rescaled accuracy score of each link S'_a on belief-speaking similarity D'_b and fact-speaking similarity D'_f in tweet texts, with party P as fixed variable following Eq.(1). The table reports results for the fixed effects. 442,500 observations were included. Regression was performed with the function `lmer` from the R library lme4[16]. No adjustments were made for multiple comparisons.

	coef.	std. err.	t	$P > \|t\|$	[0.025	0.975]
Intercept	0.8148	0.0026	308.592	$< 10^{-16}$	0.8097	0.8200
D'_b	-0.1198	0.0088	-13.577	$< 10^{-16}$	-0.1371	-0.1025
D'_f	0.0967	0.0090	10.692	$< 10^{-16}$	0.0790	0.1145
Republican	-0.0711	0.0038	-18.784	$< 10^{-16}$	-0.0785	-0.0637
$D'_b \times D'_f$	-0.0293	0.0176	-1.662	0.0972	-0.0638	0.0052
$D'_b \times$ Republican	-0.0590	0.0134	-4.411	$1.2 \cdot 10^{-5}$	-0.0852	-0.0328
$D'_f \times$ Republican	0.0397	0.0138	2.885	0.0041	0.0127	0.0667
$D'_b \times D'_f \times$ Republican	-0.1964	0.0268	-7.340	$6.9 \cdot 10^{-13}$	-0.2489	-0.1440
Observations		442500	AIC			-399392
Marginal R^2		0.049	log-Likelihood			199715
Conditional R^2		0.179	BIC			-399184

Supplementary Table 4 Results of a linear mixed effects model for the dependence of the rescaled transparency score of each link S'_t on the belief-speaking similarity D'_b and fact-speaking similarity D'_f in tweet texts, with party P as fixed variable following Eq.(2). The table reports results for the fixed effects. 442,500 observations were included. Regression was performed with the function `lmer` from the R library lme4[16]. No adjustments were made for multiple comparisons.

	coef.	std. err.	t	$P > \|t\|$	[0.025	0.975]
Intercept	0.9585	0.0025	380.958	$< 10^{-16}$	0.9536	0.9634
D'_b	-0.0631	0.0081	-7.804	$2.9 \cdot 10^{-14}$	-0.0789	-0.0473
D'_f	0.0646	0.0084	7.638	$9.5 \cdot 10^{-14}$	0.0481	0.0811
Republican	-0.0944	0.0036	-26.178	$< 10^{-16}$	-0.1015	-0.0874
$D'_b \times D'_f$	-0.0382	0.0165	-2.339	0.0207	-0.0705	-0.0059
$D'_b \times$ Republican	-0.0859	0.0123	-6.982	$7.5 \cdot 10^{-12}$	-0.1100	-0.0618
$D'_f \times$ Republican	0.0458	0.0129	3.565	0.0004	0.0206	0.0711
$D'_b \times D'_f \times$ Republican	-0.1901	0.0252	-7.560	$1.4 \cdot 10^{-13}$	-0.2394	-0.1408
Observations		442500	AIC			-400759
Marginal R^2		0.080	log-Likelihood			200399
Conditional R^2		0.196	BIC			-400551

database as well (Krippendorff's α of 0.84). This is also shown in a recent preprint[15] that compares both data bases.

S8 Independent list of untrustworthy sources

We compiled a list of trustworthiness ratings from a range of academic sources and fact-checking sites. Most of these sources were also used by [17]. The list includes Bufale [18], Bufalopedia [19], Butac [20], Buzzfeed News [21], Columbia Journalism Review [22], Fake News Watch [23], Media Bias Fact Check [24], Politifact [25], and Melissa Zimdars [26]. After removing duplicates, our list contained 4,767 domains, 1,677 of which were also contained in the NewsGuard data base, as of March 1, 2022.

The main challenge in combining lists from different fact checkers lies in unifying the labels the fact checkers assign to the domains. To address this, we devised a scheme where we rated each domain on two dimensions that we consider to be important to assess reliability and trustworthiness of information: "accuracy" and "transparency". We devise an accuracy score S_{a} that varies from 1 (false information) to 5 (scientific) and a transparency score S_{t} that varies from 1 (no transparency) to 3 (transparent). We provide a more detailed description of the five accuracy and three transparency levels in Tables Supplementary Table 5 and Supplementary Table 6. Mappings of the labels of individual fact checking sites to accuracy and transparency scores as well as the full list of domains are provided at [27].

Supplementary Table 5 Description of accuracy scores.

Score	Label	Description
1	False Information	No or very little accuracy (e.g. fake news, conspiracy, satire)
2	Clickbait	Might contain smatterings of facts but is mostly misleading or clickbait
3	Biased	Mixed accuracy, half-truths, left/right bias
4	Mainstream	Low bias, mainstream media
5	Scientific	No reporting bias, scientific information

Supplementary Table 6 Description of transparency scores.

Score	Label	Description
1	No Transparency	Intentionally misleading or no information about editorial process (e.g. fake news, conspiracy)
2	Mixed Transparency	Sites with (partially) transparent intention, but can still be misunderstood because of the way articles are written (e.g. bias, clickbait, satire)
3	Transparent	Sites with a transparent editorial process and legal notice (e.g. mainstream, scientific news)

After mapping all individual lists to the accuracy and transparency dimensions, we label every domain that has an accuracy score of 1 (False Information) or 2 (Clickbait) and/or a transparency score of 1 (No Transparency) as "unreliable". This results in a total of 2,170 domains being labelled as "unreliable" and 2,597 as "reliable". For the 1,677 domains that are contained in both data bases, the Krippendorff's α between "untrustworthy" (score < 60 in NewsGuard) and "unreliable" in the independently compiled data base is 0.84, which shows a very high agreement between the two databases. The independently compiled domain list including the unified labels is openly accessible at https://doi.org/10.5281/zenodo.6536692.

After excluding links to other social media platforms (e.g., twitter.com, facebook.com, youtube.com, and instagram.com) as well as links to search services (google.com, yahoo.com), the database covers a very similar share of links as the NewsGuard data base (between 20% and 60%) — see also Extended Data Figure 3 **B** in the main article.

S9 Honesty components by state

To examine geographical heterogeneity, we averaged NewsGuard scores across representatives and senators within each state, broken down by party. The results are shown in Figure Supplementary Figure 10, plotting each state's NewsGuard score against average belief-speaking similarity (left panels) and fact-speaking similarity (right panels), respectively. The size of plotting symbols additionally represents the vote share for Trump (in the bottom panels) and for Biden (top panels) during the 2020 presidential election. It can be seen that quality of information being shared by Republicans tends to be lower in southern states (e.g., AL, TN, TX, OK, KY) than in the north (e.g., NH, AK, ME). For democrats, no clearly discernible pattern emerges.

We also considered the outcome of the 2020 presidential election and compared the states that were called for Trump and Biden, respectively. In states that were called for Biden, Democrat members of Congress on average have a NewsGuard score of 94.5 whereas Republicans have 88.6. In states that were called for Trump, the NewsGuard scores were 94.2 (Democrats) and 87.7 (Republicans), respectively. These differences were small, suggesting that the electoral pattern in their home states did not affect the quality of information shared by members of Congress.

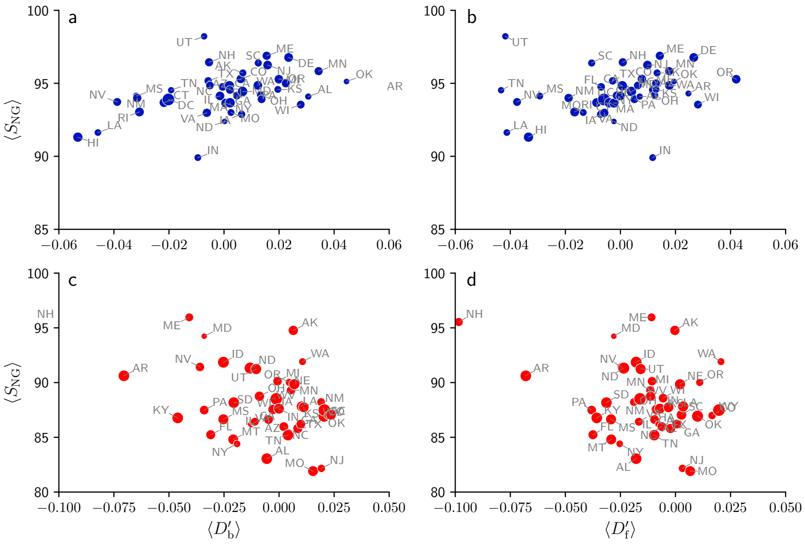

Supplementary Figure 10 Honesty components by state. Panels a and c show the NewsGuard score S_{NG} over belief-speaking similarity D'_{b} averaged by state for Democratic and Republican members of Congress, respectively. Panels b and d show S_{NG} over D'_{f} averaged by state for Democratic and Republican members of Congress, respectively. Marker sizes are scaled with the percentage of votes for Biden in the 2020 presidential election for the panels showing Democratic Congress members, and with the percentage of votes for Trump in the panels showing Republican Congress members. Note that the axes are scaled separately for each panel to reduce visual density of the point cloud.

S10 Mediation analysis

Why is it the case that belief speaking is the preferred means to spread low-quality information? One possibility is that belief-speaking is the result of Republican politicians' desire to disparage Democrats, as suggested by [28], given that belief speaking was found to be associated with greater negative sentiment (see Figure Supplementary Figure 7), and given that lower-quality information tends to be biased towards negativity[29]. According to this theory, the relationship between belief-speaking and low-quality shared information should be mediated by negative sentiment. On the other hand, if belief speaking were involved in the dissemination of poor quality content for other reasons, it should mediate the association involving negative sentiment.

To test these opposing predictions, we examined separately for Democrats and Republicans whether (1) negative sentiment mediated the effects of belief speaking on sharing low-quality information, or (2) belief speaking mediated the effects of negative sentiment on sharing low-quality information. For each user, we computed mean scores of negative sentiment (measured via VADER, see Section "VADER text analysis"), belief speaking similarity, and prevalence of sharing low-quality news (average NewsGuard score of the shared articles). We conducted a causal mediation analysis using the 'mediation' R package[30] and a bootstrap method with 10,000 iterations.

Among Republicans, when considering negative sentiment as a mediator, the effect of the direct path was not statistically significant (mean direct effect $= -9.56$, 95% CI of bootstrapped samples $= [-21.35, 1.10]$, $p = .077$). The mediation, however, was significant (average causal mediation effect $= -19.48$, 95% CI $= [-26.92, -13.43]$, $p < .001$), accounting for 67% of the total effect. When considering belief speaking as a mediator, the opposite pattern emerged: the direct effect was statistically significant (mean direct effect $= -125.87$, 95% CI $= [-159.83, -93.33]$, $p = .001$), but the average causal mediation effect did not reach statistical significance, (average causal mediation effect $=$ -11.15, 95% CI $= [-24.08, 0.83]$, $p = .067$). See Tables Supplementary Table 7 and Supplementary Table 8 for the full details. These results align with the findings of [28], suggesting that the relationship between belief-speaking and low-quality shared information is indeed driven by negative sentiment.

Supplementary Table 7 Mediation analysis with belief speaking similarity as mediator. ACME = average causal mediation effect; ADE = average direct effect. 519 observations were included for Republicans and 525 for Democrats. Mediation analysis was performed using the function `mediate` from the R package mediation, version 4.5.0. No adjustments were made for multiple comparisons.

	Parameter	Estimate	*P*	[0.025	0.975]
Republicans	ACME	-11.146	0.067	-24.084	0.830
	ADE	-125.870	$<.001$	-159.827	-93.327
	Total Effect	-137.016	$<.001$	-168.150	-108.366
	Prop. Mediated	0.0813	0.067	-0.006	0.185
Democrats	ACME	3.985	0.074	-0.387	9.366
	ADE	6.045	0.287	-5.020	17.609
	Total Effect	10.030	0.047	0.123	20.331
	Prop. Mediated	0.397	0.119	-0.236	2.619

Supplementary Table 8 Mediation analysis with negative sentiment as mediator. ACME = average causal mediation effect; ADE = average direct effect. 519 observations were included for Republicans and 525 for Democrats. Mediation was performed using the function `mediate` from the R package mediation, version 4.5.0. No adjustments were made for multiple comparisons.

	Parameter	Estimate	*P*	[0.025	0.975]
Republicans	ACME	-19.480	$<.001$	-26.916	-13.433
	ADE	-9.558	0.077	-21.352	1.097
	Total Effect	-29.038	$<.001$	-42.276	-18.018
	Prop. Mediated	0.671	$<.001$	0.442	1.055
Democrats	ACME	0.698	0.290	-0.655	2.462
	ADE	3.518	0.0732	-0.297	8.682
	Total Effect	4.216	0.007	0.924	8.990
	Prop. Mediated	0.166	0.296	-0.162	1.158

S11 Robustness analysis using only a restricted number of tweets per account

The number of tweets posted by an individual account varies widely: while the median number of tweets posted by an account is 2876, the mean is 4278, with the most prolific account posting 52,055 tweets and 10% of the accounts posting 9800 tweets or more in the observed time span (November 6, 2010 to December 31, 2022).

To assess whether our results are driven by accounts that post a large number of tweets, we repeat our main analysis analysis reported in Figure 3, including only the latest 3200 tweets from every account. The results of fitting the linear mixed effects model following Eq. (1) in Table Supplementary Table 9 show only minute deviations from the results presented in the main text where we used all tweets to fit the model (see Extended Data Table 2).

Supplementary Table 9 Results of a linear mixed effects model for the dependence of the rescaled NewsGuard score of each link S'_{NG} on belief-speaking similarity D'_{b} and fact-speaking similarity D'_{f} in tweets, with party P as fixed variable following Eq.(1). The table reports results for the fixed effects. Observations were restricted to the latest 3200 tweets for every accounts. A total of 247,947 observations were included. Regression was performed with the function `lmer` from the R library lme4 [16]. No adjustments were made for multiple comparisons.

	coef.	std. err.	t	$P > \|t\|$	[0.025	0.975]
Intercept	0.9443	0.0016	584.076	$< 10^{-16}$	0.9411	0.9475
D'_{b}	-0.0002	0.0064	0.030	0.9763	-0.0124	0.0128
D'_{f}	0.0165	0.0064	2.582	0.0101	0.0040	0.0290
Republican	-0.0647	0.0024	-27.293	$< 10^{-16}$	-0.0694	-0.0601
$D'_{\mathrm{b}} \times D'_{\mathrm{f}}$	0.0062	0.0139	0.447	0.6548	-0.0211	0.0335
D'_{b} × Republican	-0.1372	0.0098	-13.966	$< 10^{-16}$	-0.1564	-0.1179
D'_{f} × Republican	0.0794	0.0098	8.114	$2.2 \cdot 10^{-15}$	0.0602	0.0986
$D'_{\mathrm{b}} \times D'_{\mathrm{f}}$ × Republican	-0.1732	0.0200	-8.664	$< 10^{-16}$	-0.2124	-0.1340
Observations		247947	AIC			-388518
Marginal R^2		0.081	log-Likelihood			194278
Conditional R^2		0.174	BIC			-388320

S12 Increase of belief-speaking and fact-speaking similarity by account

To investigate the overall increase of both belief-speaking and fact-speaking reported in Fig. 2 in the main text, we investigated which politicians contributed most to the overall increase in both honesty components. We show the top 10 accounts with the largest change in belief-speaking and fact-speaking similarity between the 2010–2013 and the 2019–2022 period for both Democrats and Republicans in Tables Supplementary Table 10 and Supplementary Table 11.

Supplementary Table 10 Twitter accounts of Democratic and Republican representatives with the highest increase in average belief-speaking similarity $\langle D'_{\mathrm{b}} \rangle_{\mathrm{acc}}$ between the period 2011–2013 and 2019–2022.

account handle	$\langle D'_{\mathrm{b}} \rangle_{\mathrm{acc}}$ 2010–2013	$\langle D'_{\mathrm{b}} \rangle_{\mathrm{acc}}$ 2019–2022	difference
Democrats			
SenatorLujan	-0.43	0.01	0.44
SenStabenow	-0.33	0.04	0.36
SenBooker	0.24	0.04	0.28
aguilarpete	-0.01	0.21	0.22
WilliamKeating	-0.17	0.03	0.21
USRepKeating	-0.16	0.04	0.20
pallonefornj	-0.14	0.05	0.19
BobbyScott4VA3	-0.28	-0.10	0.18
TulsiPress	-0.15	0.03	0.17
Matsui4Congress	-0.16	0.01	0.17
Republicans			
SenBobCorker	-0.14	0.16	0.31
GrassleyPress	-0.28	0.00	0.29
McCaulforTexas	-0.25	0.02	0.26
krhern	-0.15	0.09	0.24
votetimscott	-0.12	0.09	0.21
MaElviraSalazar	-0.51	-0.31	0.20
congbillposey	-0.31	-0.12	0.19
MacTXPress	-0.12	0.07	0.19
MikeKellyforPA	-0.13	0.06	0.19
JohnKennedyLA	-0.12	0.07	0.19

Supplementary Table 11 Twitter accounts of Democratic and Republican representatives with the highest increase in average fact-speaking similarity $\langle D'_\mathrm{f} \rangle_\mathrm{acc}$ between the period 2011–2013 and 2019–2022.

account handle	$\langle D'_\mathrm{f} \rangle_\mathrm{acc}$ 2010–2013	$\langle D'_\mathrm{f} \rangle_\mathrm{acc}$ 2019–2022	difference
	Democrats		
SenatorLujan	-0.33	0.02	0.35
SenStabenow	-0.25	0.04	0.29
WilliamKeating	-0.19	0.01	0.20
TulsiPress	-0.14	0.05	0.19
DeGette5280	-0.15	0.03	0.18
USRepKeating	-0.14	0.04	0.18
Matsui4Congress	-0.18	-0.02	0.16
SenBooker	-0.12	0.04	0.16
RepJoseSerrano	-0.21	-0.06	0.15
BobbyScott4VA3	-0.25	-0.11	0.15
	Republicans		
McCaulforTexas	-0.25	0.00	0.26
SenBobCorker	-0.11	0.12	0.23
GrassleyPress	-0.17	0.04	0.21
TeamCMR	-0.14	0.05	0.19
congbillposey	-0.25	-0.06	0.19
cindyhydesmith	-0.14	0.03	0.16
stephaniebice	-0.15	0.01	0.16
votetimscott	-0.13	0.03	0.16
CurtisUT	-0.13	0.03	0.16
MikeKellyforPA	-0.15	0.01	0.15

S13 Robustness analysis using different embeddings

In addition to GloVe[31] embeddings used for the results presented in the main text, we also calculated D'_{b} and D'_{f} using word2vec[32] and fasttext[33] embeddings to exclude a dependence of our results on the choice of embedding. We note that both GloVe and fasttext were trained on the "common crawl" corpus, whereas word2vec was trained on Google news, a corpus with a more restricted scope. Results for the linear mixed effects modeling following Eq. (1) using word2vec and fasttext embeddings are shown in Tables Supplementary Table 12 and Supplementary Table 13, respectively. Results for both word2vec and fasttext are similar to the results using GloVe reported in Extended Data Table 2.

Supplementary Table 12 Results of a linear mixed effects model for the dependence of the rescaled NewsGuard score of each link S'_{NG} on belief-speaking similarity D'_{b} and fact-speaking similarity D'_{f} in tweets, with party P as fixed variable following Eq.(1). In contrast to Tables Supplementary Table 9 and Extended Data Tables 2 and 3 in the main text, the belief-speaking and fact-speaking similarities have been calculated using word2vec[32] embeddings. The table reports results for the fixed effects. A total of 504,809 observations were included. Regression was performed with the function `lmer` from the R library lme4[16]. No adjustments were made for multiple comparisons.

	coef.	std. err.	t	$P > \|t\|$	[0.025	0.975]
Intercept	0.9435	0.0016	592.953	$< 10^{-16}$	0.9403	0.9466
D'_{b}	0.0038	0.0088	0.430	0.6672	-0.0135	-0.0211
D'_{f}	0.0243	0.0087	2.804	0.0052	0.0073	0.0413
Republican	-0.0671	0.0023	-29.463	$< 10^{-16}$	-0.0716	-0.0627
$D'_{\mathrm{b}} \times D'_{\mathrm{f}}$	0.0061	0.0158	0.383	0.7018	-0.0250	0.0371
D'_{b} × Republican	-0.2043	0.0131	-15.590	$< 10^{-16}$	-0.2300	-0.1787
D'_{f} × Republican	0.1031	0.0130	7.911	$1.2 \cdot 10^{-14}$	0.0775	0.1286
$D'_{\mathrm{b}} \times D'_{\mathrm{f}}$ × Republican	-0.2765	0.0239	-11.580	$< 10^{-16}$	-0.3233	-0.2297
Observations		504809	AIC			-801648
Marginal R^2		0.087	log-Likelihood			400843
Conditional R^2		0.184	BIC			-801436

Supplementary Table 13 Results of a linear mixed effects model for the dependence of the rescaled NewsGuard score of each link S'_{NG} on belief-speaking similarity D'_{b} and fact-speaking similarity D'_{f} in tweets, with party P as fixed variable following Eq.(1). In contrast to Tables Supplementary Table 9 and Extended Data Tables 2 and 3 in the main text, the belief-speaking and fact-speaking similarities have been calculated using fasttext[33] embeddings. The table reports results for the fixed effects. A total of 504,809 observations were included. Regression was performed with the function `lmer` from the R library lme4[16]. No adjustments were made for multiple comparisons.

	coef.	std. err.	t	$P > \|t\|$	[0.025	0.975]
Intercept	0.9438	0.0016	577.940	$< 10^{-16}$	0.9406	0.9470
D'_{b}	0.0093	0.0081	1.151	0.251	-0.0065	0.0252
D'_{f}	-0.0061	0.0076	-0.793	0.427	-0.0210	0.0089
Republican	-0.0716	0.0023	-30.611	$< 10^{-16}$	-0.0762	-0.0670
$D'_{\mathrm{b}} \times D'_{\mathrm{f}}$	0.0452	0.0486	0.929	0.353	-0.0501	0.1405
$D'_{\mathrm{b}} \times$ Republican	-0.1919	0.0122	-15.736	$< 10^{-16}$	-0.2158	-0.1680
$D'_{\mathrm{f}} \times$ Republican	0.0796	0.0116	6.836	$2.0 \cdot 10^{-11}$	0.0568	0.1024
$D'_{\mathrm{b}} \times D'_{\mathrm{f}} \times$ Republican	-0.0730	0.0751	-0.972	0.331	-0.2201	0.0742
Observations		504809	AIC			-799568
Marginal R^2		0.086	log-Likelihood			399803
Conditional R^2		0.182	BIC			-799358

S14 Increase of belief-speaking and fact-speaking similarity by keyword

To asses which keywords in the belief-speaking and fact-speaking dictionaries contributed most to the increase of overall belief-speaking and fact-speaking similarity, we created embeddings of single keywords to calculate the centered and length-corrected similarity D'_{kw} of tweets to a given keyword. For every keyword, we then calculated the mean similarity for tweets from the years 2010 to 2013 and for tweets from the years 2019 to 2022. We show the increase in similarity for every keyword in Figure Supplementary Figure 11.

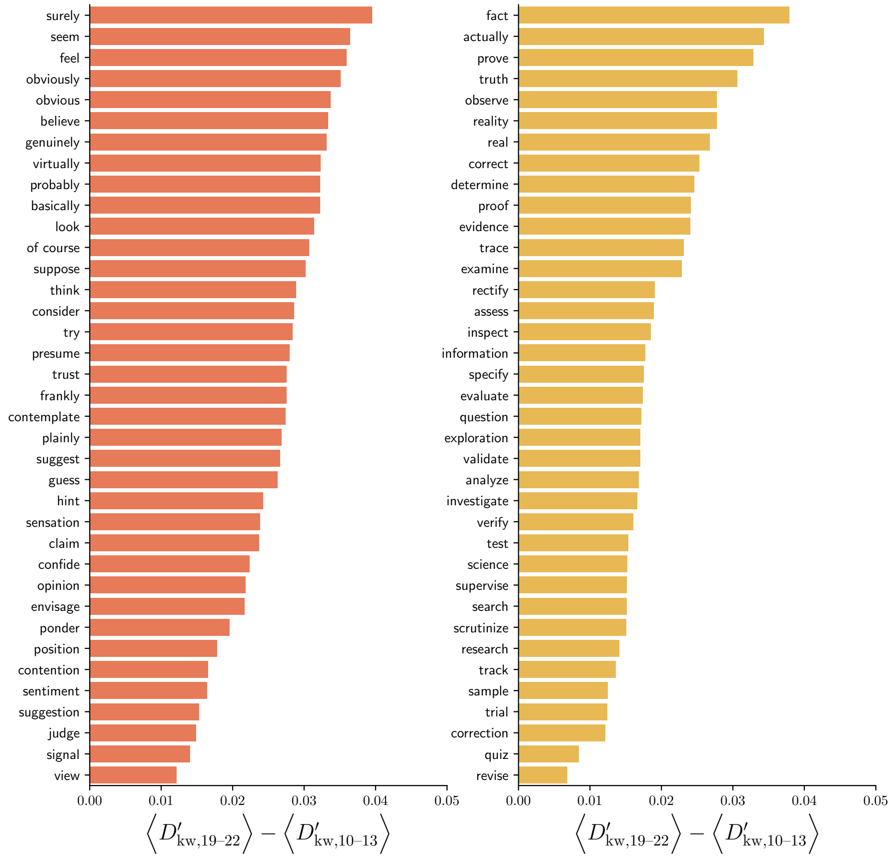

Supplementary Figure 11 Increase of similarity score of tweets of individual keywords from the belief-speaking (left) and fact-speaking (right) dictionaries between the time-periods 2010–2013 and 2019–2022.

References

[1] S. Palan, C. Schitter, Prolific.ac – a subject pool for online experiments. Journal of Behavioral and Experimental Finance **17**, 22–27 (2018). https://doi.org/10.1016/j.jbef.2017.12.004

[2] C. Hutto, E. Gilbert, VADER: A parsimonious rule-based model for sentiment analysis of social media text. Proceedings of the International AAAI Conference on Web and Social Media **8**, 216–225 (2014). https://doi.org/10.1609/icwsm.v8i1.14550

[3] R.L. Boyd, A. Ashokkumar, S. Seraj, J.W. Pennebaker, The development and psychometric properties of LIWC-22. Tech. rep., University of Texas at Austin, Austin, TX (2022)

[4] M.L. Newman, J.W. Pennebaker, D.S. Berry, J.M. Richards, Lying Words: Predicting Deception from Linguistic Styles. Personality and Social Psychology Bulletin **29**, 665–675 (2003). https://doi.org/10.1177/0146167203029005010

[5] J.W. Pennebaker, C.K. Chung, J. Frazee, G.M. Lavergne, D.I. Beaver, When Small Words Foretell Academic Success: The Case of College Admissions Essays. PloS one **9**, e115,844 (2014). https://doi.org/10.1371/journal.pone.0115844

[6] W.J. Brady, M.J. Crockett, J.J.V. Bavel, The MAD Model of Moral Contagion: The Role of Motivation, Attention, and Design in the Spread of Moralized Content Online. Perspectives on Psychological Science **15**, 978–1010 (2020). https://doi.org/10.1177/1745691620917336

[7] M. Grootendorst, BERTopic: Neural topic modeling with a class-based TF-IDF procedure. arXiv (2022). https://doi.org/10.48550/arXiv.2203.05794

[8] L. McInnes, J. Healy, J. Melville, UMAP: Uniform manifold approximation and projection for dimension reduction. arXiv (2018). https://doi.org/10.48550/arXiv.1802.03426

[9] R.J. Campello, D. Moulavi, J. Sander, in *Pacific-Asia conference on knowledge discovery and data mining* (Springer, 2013), pp. 160–172. https://doi.org/10.1007/978-3-642-37456-2_14

[10] R. Egger, J. Yu, A Topic Modeling Comparison Between LDA, NMF, Top2Vec, and BERTopic to Demystify Twitter Posts. Frontiers in Sociology **7** (2022). https://doi.org/10.3389/fsoc.2022.886498

[11] F. Alhaj, A. Al-Haj, A. Sharieh, R. Jabri, Improving Arabic cognitive distortion classification in Twitter using BERTopic. International Journal of Advanced Computer Science and Applications **13**, 854–860 (2022). https://doi.org/10.14569/IJACSA.2022.0130199

[12] M. Nikita, M.M. Nikita. Package 'ldatuning' (2016). URL https://CRAN.R-project.org/package=ldatuning

[13] M. Cinelli, G. De Francisci Morales, A. Galeazzi, W. Quattrociocchi, M. Starnini, The echo chamber effect on social media. Proceedings of the National Academy of Sciences **118**, e2023301,118 (2021). https://doi.org/10.1073/pnas.2023301118

[14] Pew Research Center. https://www.pewresearch.org/ (2022). Accessed 2022-06-15

[15] H. Lin, J. Lasser, S. Lewandowsky, R. Cole, A. Gully, D.G. Rand, G. Pennycook, High level of agreement across different news domain quality ratings. PsyArXiv (2022). https://doi.org/10.31234/osf.io/qy94s

[16] D. Bates, M. Mächler, B. Bolker, S. Walker, Fitting linear mixed-effects models using lme4. Journal of Statistical Software **67**, 1–48 (2015). https://doi.org/10.18637/jss.v067.i01

[17] R. Gallotti, F. Valle, N. Castaldo, P. Sacco, M. De Domenico, Assessing the risks of 'infodemics' in response to COVID-19 epidemics. Nature Human Behaviour **4**, 1285–1293 (2020). https://doi.org/10.1038/s41562-020-00994-6

[18] Bufale. https://www.bufale.net/. Accessed: 2022-05-01

[19] Bufalopedia. Un catalogo di indagini e risorse antibufala. https://bufalopedia. blogspot.com/p/siti-creatori-di-bufale.html. Accessed: 2022-05-01

[20] Butac. The black list. https://www.butac.it/the-black-list/. Accessed: 2022-05-01

[21] Buzzfeed News. Inside the partisan fight for your news feed. https://www.buzzfeednews.com/article/craigsilverman/inside-the-partisan-fight-for-your-news-feed. Accessed: 2022-05-01

[22] Columbia Journalism Review. CJR index of fake-news, clickbait, and hate. http://web.archive.org/web/20210720140548. Accessed: 2022-05-01

[23] Fake News Watch. https://web.archive.org/web/20180213181029. Accessed: 2022-05-01

[24] Media Bias Fact Check. Media bias fact check. https://mediabiasfactcheck.com. Accessed: 2022-05-01

[25] Politifact. Politifact's guide to fake news websites and what they peddle. https://www.politifact.com/article/2017/apr/20/politifacts-guide-fake-news-websites-and-what-they. Accessed: 2022-05-01

[26] M. Zimdars. My "fake news list" went viral. but made-up stories are only part of the problem. https://www.washingtonpost.com/posteverything/wp/2016/11/18/my-fake-news-list-went-viral-but-made-up-stories-are-only-part-of-the-problem. Accessed: 2022-05-01

[27] J. Lasser. List of domain accuracy and transparency scores v1.1 (2022). https://doi.org/10.5281/ZENODO.6536692

[28] M. Osmundsen, A. Bor, P.B. Vahlstrup, A. Bechmann, M.B. Petersen, Partisan Polarization Is the Primary Psychological Motivation behind Political Fake News Sharing on Twitter. American Political Science Review **115**, 999–1015 (2021). https://doi.org/10.1017/S0003055421000290

[29] S. Soroka, P. Fournier, L. Nir, Cross-national evidence of a negativity bias in psychophysiological reactions to news. Proceedings of the National Academy of Sciences **116**, 18,888–18,892 (2019). https://doi.org/10.1073/pnas.1908369116

[30] D. Tingley, T. Yamamoto, K. Hirose, L. Keele, K. Imai, Mediation: R package for causal mediation analysis. Tech. rep., UCLA Statistics/American Statistical Association (2014)

[31] J. Pennington, R. Socher, C.D. Manning, in *Proceedings of the 2014 conference on empirical methods in natural language processing (EMNLP)* (2014), pp. 1532–1543. https://doi.org/10.3115/v1/D14-1162

[32] T. Mikolov, K. Chen, G. Corrado, J. Dean, Efficient estimation of word representations in vector space. arXiv (2013). https://doi.org/10.48550/arXiv.1301.3781Focustolearnmore

[33] P. Bojanowski, E. Grave, A. Joulin, T. Mikolov, Enriching Word Vectors with Subword Information. Transactions of the Association for Computational Linguistics **5**, 135–146 (2017). https://doi.org/10.1162/tacl_a_00051
