# Supplementary material for: From alternative conceptions of honesty to alternative facts in communications by US politicians
Source: Nat Hum Behav. 2023 Sep 25;7(12):2140–51. doi: 10.1038/s41562-023-01691-w (PMC10730411; doi:10.1038/s41562-023-01691-w)
Supplement: Supplementary file 1 [file 41562_2023_1691_MOESM2_ESM.pdf]

nature portfolio

Corresponding author(s): Stephan Lewandowsky

Last updated by author(s): Jul 17, 2023

Reporting Summary

Nature Portfolio wishes to improve the reproducibility of the work that we publish. This form provides structure for consistency and transparency in reporting. For further information on Nature Portfolio policies, see our Editorial Policies and the Editorial Policy Checklist.

Statistics

For all statistical analyses, confirm that the following items are present in the figure legend, table legend, main text, or Methods section.

n/a	Confirmed	
☐	☒	The exact sample size (*n*) for each experimental group/condition, given as a discrete number and unit of measurement
☐	☒	A statement on whether measurements were taken from distinct samples or whether the same sample was measured repeatedly
☐	☒	The statistical test(s) used AND whether they are one- or two-sided<br>*Only common tests should be described solely by name; describe more complex techniques in the Methods section.*
☐	☒	A description of all covariates tested
☐	☒	A description of any assumptions or corrections, such as tests of normality and adjustment for multiple comparisons
☐	☒	A full description of the statistical parameters including central tendency (e.g. means) or other basic estimates (e.g. regression coefficient) AND variation (e.g. standard deviation) or associated estimates of uncertainty (e.g. confidence intervals)
☐	☒	For null hypothesis testing, the test statistic (e.g. *F*, *t*, *r*) with confidence intervals, effect sizes, degrees of freedom and *P* value noted<br>*Give P values as exact values whenever suitable.*
☒	☐	For Bayesian analysis, information on the choice of priors and Markov chain Monte Carlo settings
☒	☐	For hierarchical and complex designs, identification of the appropriate level for tests and full reporting of outcomes
☐	☒	Estimates of effect sizes (e.g. Cohen's *d*, Pearson's *r*), indicating how they were calculated

Our web collection on statistics for biologists contains articles on many of the points above.

Software and code

Policy information about availability of computer code

Data collection	Data was collected with custom Python (v3.9.1) and R (v4.2) scripts. Specifically, the third-party Python packages twarc2 (v2.13.0) and newspaper3k (v0.2.8) were used. Code for data collection is available under accession code 10.5281/zenodo.7723109.
Data analysis	Data was analysed with custom Python (v3.9.1) and R (v4.2) scripts. Specifically, the third-party Python packages statsmodels (v0.13.2), scipy (v1.7.3), pengouin (v0.5.2), sentence transformers (v2.2.2) and torch (v1.8.1+cu102) and the third-party R package lme4 (v1.1-34) were used. Code for data analysis is available under accession code 10.5281/zenodo.7723109.

For manuscripts utilizing custom algorithms or software that are central to the research but not yet described in published literature, software must be made available to editors and reviewers. We strongly encourage code deposition in a community repository (e.g. GitHub). See the Nature Portfolio guidelines for submitting code & software for further information.

Data

Policy information about availability of data

All manuscripts must include a data availability statement. This statement should provide the following information, where applicable:

- Accession codes, unique identifiers, or web links for publicly available datasets
- A description of any restrictions on data availability
- For clinical datasets or third party data, please ensure that the statement adheres to our policy

The lists of Twitter handles of members of congress used to build the tweet corpus are available from www.socialseer.com (114th and 115th Congress), https://

doi.org/10.7910/DVN/MBOJNS (116th Congress), and https://triagecancer.org/congressional-social-media (117th and 118th Congress).The tweet IDs of the tweet texts and URLs of the articles analysed in this study are deposited in OSF under accession code https://doi.org/10.17605/OSF.IO/VNY8K. Dictionaries of keywords associated with the different conceptions of honestyare deposited in OSF under accession code https://doi.org/10.17605/OSF.IO/VNY8K. The independently compiled list of domain accuracy and transparency scores is deposited on GitHub under accession code https://doi.org/10.5281/zenodo.6536692.The NewsGuard data base used to asses domain trustworthiness is commercially available from NewsGuard and cannot be shared publicly.Aggregated values for information trustworthiness and honesty components for tweets and articles used to produce all figures in this article are deposited in OSF under accession code https://doi.org/10.17605/OSF.IO/VNY8K.

Research involving human participants, their data, or biological material

Policy information about studies with human participants or human data. See also policy information about sex, gender (identity/presentation), and sexual orientation and race, ethnicity and racism.

Reporting on sex and gender	N/A
Reporting on race, ethnicity, or other socially relevant groupings	N/A
Population characteristics	N/A
Recruitment	N/A
Ethics oversight	N/A

Note that full information on the approval of the study protocol must also be provided in the manuscript.

Field-specific reporting

Please select the one below that is the best fit for your research. If you are not sure, read the appropriate sections before making your selection.

☐ Life sciences ☒ Behavioural & social sciences ☐ Ecological, evolutionary & environmental sciences

For a reference copy of the document with all sections, see nature.com/documents/nr-reporting-summary-flat.pdf

Behavioural & social sciences study design

All studies must disclose on these points even when the disclosure is negative.

Study description	Quantitative text analysis from social media and news.
Research sample	The Twitter accounts of U.S. Congress Members that were analysed in this study were compiled from public sources, e.g. www.socialseer.com (114th and 115th Congress), https://doi.org/10.7910/DVN/MBOJNS (116th Congress), and https://triagecancer.org/congressional-social-media (117th and 118th Congress).
Sampling strategy	The Twitter accounts included in this study are a comprehensive collection of Twitter accounts of U.S. Congress people that were active during the observation period 2011-2023. No sampling was involved in the compilation of Twitter accounts.
Data collection	Data retrieval through the Twitter API and The New York Times API. Analysis of the COHA corpus.
Timing	Twitter data was retrieved on February 12, 2023. The data spans a period between January 2011 and February 2023.
Data exclusions	Retweets, non-English tweets, duplicatet tweets were excluded. Only tweets from Democrat and Republican Congress Members were included.
Non-participation	US Congress Members who deleted their account or made it private prior to February 12, 2023 are self-excluded from the analysis.
Randomization	No randomization was performed, this is a purely observational study.

Reporting for specific materials, systems and methods

We require information from authors about some types of materials, experimental systems and methods used in many studies. Here, indicate whether each material, system or method listed is relevant to your study. If you are not sure if a list item applies to your research, read the appropriate section before selecting a response.

Materials & experimental systems

n/a	Involved in the study
☒	☐ Antibodies
☒	☐ Eukaryotic cell lines
☒	☐ Palaeontology and archaeology
☒	☐ Animals and other organisms
☒	☐ Clinical data
☒	☐ Dual use research of concern
☒	☐ Plants

Methods

n/a	Involved in the study
☒	☐ ChIP-seq
☒	☐ Flow cytometry
☒	☐ MRI-based neuroimaging
